# ROHHAD syndrome: an interdisciplinary perspective

**DOI:** 10.3389/fped.2026.1777286

**Published:** 2026-06-03

**Authors:** Anna Mercante, Annalisa Salerno, Anna Marinetto, Anna Santini, Benedetta Tascini, Alessia Raffagnato, Valentina De Tommasi, Antuan Divisic, Franca Benini

**Affiliations:** 1 Department of Biomedical and Neuromotor Sciences (DIBINEM), University of Bologna, Bologna, Italy; 2 Department of Women's and Children's Health, Pediatric Pain and Palliative Care Service, University Hospital of Padua, Padua, Italy; 3 Department of Medicine, University of Padua, Padua, Italy; 4 Healthcare Profession Department, University Hospital of Padua, Padua, Italy; 5 Department of Women's and Children's Health, Child and Adolescent Neuropsychiatric Unit, University Hospital of Padua, Padua, Italy; 6 Department of Women's and Children's Health, University of Padua, Padua, Italy

**Keywords:** autonomic dysregulation, complex care needs, hypothalamic dysfunction, hypoventilation, pediatric palliative care, rapid-onset obesity, ROHHAD syndrome

## Abstract

**Objective:**

To characterize the multisystem presentation and care requirements of rapid-onset obesity with hypothalamic dysfunction, hypoventilation, and autonomic dysregulation (ROHHAD) syndrome from an interdisciplinary perspective.

**Study design:**

A retrospective analysis of clinically confirmed ROHHAD cases managed within a specialized pediatric palliative care service over a 13-year period (2012–2025). Collected data included demographic and socio-familial characteristics, clinical manifestations, care needs, management strategies, disease trajectories, and outcomes. Descriptive statistics and exploratory Spearman's rank correlation were applied.

**Results:**

Six patients (three females and three males) were identified, with a median age at onset of 3.3 years and a median diagnostic delay of 1.6 years. Central hypoventilation represented a key driver of clinical complexity, with most children rapidly progressing to tracheostomy and long-term invasive ventilation. Endocrine involvement was multisystemic and frequently associated with electrolyte and metabolic instability. Autonomic dysregulation was recurrent but clinically heterogeneous. Neurodevelopmental concerns were common, and many children later developed severe behavioral dysregulation and additional neuropsychiatric symptoms that significantly affected treatment adherence and overall management. No neuroendocrine tumors were identified on the available imaging studies. At the time of data collection, four children had died, predominantly due to cardiorespiratory arrest.

**Conclusions:**

The clinical complexity of ROHHAD requires coordinated, interdisciplinary care. However, real-world data comprehensively defining patient needs and clarifying the contribution of different specialties remain limited. Our findings provide novel insights and highlight how the evolving course challenges traditional discipline-centered approaches, instead demanding ongoing, integrated collaboration to ensure adaptable, individualized care pathways.

## Introduction

Rapid-onset obesity with hypothalamic dysfunction, hypoventilation, and autonomic dysregulation (ROHHAD) syndrome is a rare and complex life-threatening disorder that predominantly affects young children (1). The exact prevalence is unknown, with only a few hundred cases documented in the literature since its formal description (2, 3). In the absence of a definitive biomarker, diagnosis is primarily clinical and relies on the exclusion of alternative conditions (4).

The characteristic initial presentation of ROHHAD is the rapid, unexplained onset of obesity in a previously healthy child, typically during early childhood (3). This progresses to global hypothalamic dysfunction, leading to endocrine and metabolic disturbances such as growth hormone deficiency, water balance abnormalities, central hypothyroidism, adrenocortical insufficiency, and pubertal disorders (5). Central hypoventilation often develops soon thereafter, manifesting as sustained alveolar hypoventilation and respiratory insufficiency. Autonomic dysregulation—affecting cardiovascular stability, gastrointestinal motility, and body temperature homeostasis—significantly increases overall morbidity (6). Notably, approximately 40%–56% of patients present with an associated neuroendocrine tumor (ROHHAD-NET), which is largely benign, though clinical presentation varies from asymptomatic to symptomatic cases (7, 8).

The etiology of ROHHAD remains unclear. While genetic studies, including whole-exome and whole-genome sequencing, have failed to identify a consistent monogenic cause (8), attention has increasingly turned toward an autoimmune mechanism. This hypothesis is corroborated by the detection of autoantibodies targeting hypothalamic and pituitary antigens in a subset of patients (9, 10).

Management is mainly supportive and symptomatic, frequently requiring coordinated interventions within specialized pediatric settings, including pediatric palliative care (PPC). Indeed, the long-term prognosis for individuals with ROHHAD is guarded, with a significant risk of mortality, most commonly due to cardiorespiratory arrest. Early recognition and integrated care are therefore crucial to optimize patient outcomes and emphasize the need for proactive vigilance (5).

Here, we present our clinical experience in managing patients with ROHHAD, thereby contributing to the existing body of knowledge on this rare condition.

## Material and methods

Applying an interdisciplinary framework, we retrospectively examined the clinical characteristics, disease progression, therapeutic needs, and management strategies of patients with ROHHAD. The study was conducted in accordance with institutional ethical standards and the Declaration of Helsinki and ARRIVE guidelines. Informed consent was obtained in accordance with local regulations. The requirement for ethics committee approval was waived given the observational nature of the study.

We reviewed the medical records of patients referred to the Pediatric Hospice of Padua from January 2012 to December 2025. Inclusion criteria were: (I) confirmed diagnosis of ROHHAD syndrome based on clinical presentation and relevant investigations; (II) pediatric age at onset; and (III) availability of comprehensive follow-up. Extracted data encompassed patient demographics and clinical history, alongside diagnostic results from laboratory, imaging, and genetic investigations. For the surviving patients (*Patients 2* and *3*), disease duration and age reflected the last available follow-up.

Due to the limited cohort size, statistical analysis was primarily descriptive. Categorical variables were expressed as frequencies and percentages, whereas continuous variables were reported as median and range owing to the non-normal data distribution. Furthermore, potential associations between age and BMI z-scores were explored using Spearman's rank correlation coefficient (*ρ*).

## Results

### Patient demographics and social history

The cohort consisted of six individuals with an equal distribution of males and females. In terms of ethnicity, four patients were Caucasian, one was of Bengali descent, and one was North African. Two children originated from families with low socioeconomic status, while the remaining four belonged to middle socioeconomic backgrounds. All families had equal access to healthcare services within the Italian national health system. Regular school attendance was limited, with only two patients attending consistently and a single child participating in extracurricular activities.

### Disease onset, diagnosis, and outcome

The median age at disease onset was 3.3 years (range 1.8–8.6, *n* = 6). Four patients had pre-existing neurodevelopmental disorders (4/6, 67%), and two of these were also born prematurely; one additional patient experienced prenatal complications. Rapid-onset obesity was the initial manifestation in most cases (5/6, 83%), except for one child who first presented with abnormalities in thirst and urine regulation and excessive sweating, prior to weight gain. Diagnostic delay ranged from 0.3 to 3.7 years, with a median of 1.6 years (*n* = 6). At the time of data collection, four out of six patients had died. Among these, the median age at death was 9.9 years (range 3.7–15.6, *n* = 4), with a median disease duration of 4.8 years (range 0.9–11.4, *n* = 4). The two surviving patients were aged 7.8 and 17.9 years at the last available follow-up, with disease durations of 5.0 and 14.2 years, respectively. To support clinical characterization, genetic testing was performed. One patient did not undergo genetic evaluation. In the remaining five, targeted PHOX2B sequencing was prioritized either by targeted Sanger sequencing or as part of whole-exome sequencing (*n* = 5). Additional investigations—including karyotyping, chromosomal microarray analysis, Prader–Willi methylation testing, and targeted mitochondrial panels—were conducted to rule out alternative syndromic and metabolic conditions. No significant genetic findings emerged, identifying only a single variant of uncertain significance (a 171 Kb duplication at 3q27.3). Similarly, radiological tumor surveillance was heterogeneous across the cohort. Urinary catecholamine excretion was generally normal; only one patient exhibited mild, transient abnormalities during the early disease phase. Comprehensive whole-body imaging was limited to three cases (PET-CT, *n* = 2; CT, *n* = 1) and was performed at a median interval of 0.6 years after disease onset (range: 0.2–1.1). The three other patients received only localized screening. Ultimately, no NETs were detected (0/6, 0%).

Table 1 provides a comprehensive overview of patient demographics and individual disease courses. See Supplementary Table S1 for detailed genetic and imaging investigations.

**Table 1:** Overview of patient demographics and disease course.

Patient	Ethnicity	Gender	Age at onset (yrs)	Previous medical history	Presenting symptom	Diagnostic delay (yrs)	Current age (yrs)^a^	Age at death (yrs)^b^	Disease duration (yrs)	NET^c^
P1	Caucasian	Male	1.8	Fetal macrocephaly, polyhydramnios	Polydipsia and polyuria with nocturia, excessive sweating	1.8	Deceased	13.2	11.4	No
P2	Caucasian	Male	2.8	Conceived by assisted reproductive technology, extreme preterm (25 w + 6 d), psychomotor delay, syringomyelia	Rapid-onset obesity	1.4	7.8	–	5.0^a^	No
P3	Caucasian	Female	3.8	Unremarkable	Rapid-onset obesity	1.1	17.9	–	14.2^a^	No
P4	North African	Female	2.8	Language delay	Rapid-onset obesity	0.3	Deceased	3.7	0.9	No
P5	Bengali	Female	4	Late preterm (34 w + 4 d), autism spectrum disorder with cognitive delay	Rapid-onset obesity	2.4	Deceased	6.7	2.6	No
P6	Caucasian	Male	8.6	Psychomotor delay with borderline cognitive functioning	Rapid-onset obesity	3.7	Deceased	15.6	7	No

D, days; NET, neuroendocrine tumor; P, patient; w, weeks.

a Calculated for surviving patients, referring to the time from symptom onset to the last follow-up (data censored).

b Calculated for deceased patients.

c As assessed by the available imaging modalities.

### Clinical features

Reflecting the diverse clinical manifestations of the disease, specific features are categorized below according to clinical domain, with their frequencies and percentages summarized in Table 2. The individual distribution of these features across the study cohort is provided in Supplementary Table S2.

**Table 2:** Distribution of clinical features across systems.

Clinical features	Patients (*n*/tot)	Percentage (%)
Endocrinological problems		
Obesity	6/6	100%
Hypogonadotropic hypogonadism	2/2	100%
Central hypothyroidism	5/6	83%
Growth hormone deficiency	3/4	75%
Adrenocortical insufficiency	4/6	67%
Hyperprolactinemia	3/5	60%
Sodium disturbances	3/5	60%
Arginine vasopressin deficiency	3/6	50%
Short stature	3/6	50%
Metabolic problems		
Hepatic steatosis	4/6	67%
Insulin resistance	2/4	50%
Impaired glucose tolerance	1/6	17%
Dyslipidemia	1/6	17%
Hepatomegaly	1/6	17%
Cholelithiasis	1/6	17%
Cholestasis	1/6	17%
Neurological problems		
Nocturnal sleep disturbances	6/6	100%
Fatigue	6/6	100%
Ocular motility disorders	5/6	83%
Excessive daytime sleepiness	4/6	67%
Motor clumsiness	4/6	67%
Ataxia	2/6	33%
Sialorrhea	2/6	33%
Dysphagia	1/6	17%
Seizures	1/6	17%
*Autonomic dysfunction*		
Decreased pain sensitivity	6/6	100%
Excessive sweating	5/6	83%
Urinary incontinence	5/6	83%
Hyperthermia^a^	4/6	67%
Bradycardia	3/6	50%
Constipation	3/6	50%
Fecal incontinence	3/6	50%
Abnormal pupillary reactivity	2/6	33%
Bilateral miosis	2/6	33%
Hypothermia^a^	1/6	17%
Cold extremities	1/6	17%
Paralytic ileus	1/6	17%
Behavioral and Psychiatric problems		
Mood disorders	5/6	83%
Psychomotor agitation	5/6	83%
Frustration intolerance	5/6	83%
Hetero-aggressive behavior	5/6	83%
Oppositional–defiant behaviors	5/6	83%
Neurocognitive dysfunction	5/6	83%
Social isolation	5/6	83%
Language decline	4/6	67%
Loss of skills and autonomy	4/6	67%
Intolerance to life-saving devices	3/6	50%
Self-injurious behavior	2/6	33%
Anxiety	1/6	17%
Respiratory problems		
Central hypoventilation	6/6	100%
Oxygen desaturations with cyanotic episodes	6/6	100%
OSAS	3/6	50%
Respiratory infections	2/6	33%
Other		
Hyperphagia	5/6	83%
Non-epileptic paroxysmal events	2/6	33%
NET^b^	0/6	0%

OSAS, obstructive sleep apnea syndrome; NET, neuroendocrine tumor.

a One patient exhibited both hyperthermia and hypothermia.

b As assessed by the available imaging modalities.

#### Endocrinological and metabolic problems

Endocrine abnormalities were documented in all cases, though presenting with variable combinations and onset timings. Central hypothyroidism was the most frequent finding (5/6, 83%), followed by central adrenal insufficiency (4/6, 67%). Within the latter group, clinical presentation was diverse, with two patients exhibiting a partial deficit and two developing the condition secondary to corticosteroid therapy. Growth hormone (GH) deficiency was identified in the majority of the tested individuals (3/4, 75%). Arginine vasopressin deficiency (AVP-D) was detected in half of the cohort (3/6, 50%). Furthermore, hyperprolactinemia was observed in three of the five patients in whom this hormone was measured (3/5, 60%); these elevations were generally mild and without evidence of macroprolactinemia. Among those screened, sodium disturbances were also frequent (3/5, 60%). The severity of these disturbances varied: one patient experienced hyponatremia followed by severe hypernatremia during the same intercurrent infectious episode, with severe hypernatremia being unresponsive to desmopressin; a second presented with severe hypernatremia at the onset of AVP-D; while the third showed only mild, transient abnormalities without sustained clinical relevance. Finally, hypogonadotropic hypogonadism was identified in both patients assessed for gonadal function (2/2, 100%); however, incomplete endocrine testing in the remaining four precluded a definitive cohort-wide assessment.

Metabolic abnormalities were variably represented. Hepatic steatosis was documented in four cases (4/6, 67%), although liver enzyme levels remained within reference ranges. Regarding glycemic control, two of the four tested patients showed evidence of insulin resistance (2/4, 50%) and one had impaired glucose tolerance (1/6, 17%), despite none developing diabetes. Further findings comprised dyslipidemia (1/6, 17%), hepatomegaly in the absence of steatosis (1/6, 17%), cholelithiasis (1/6, 17%), and cholestasis (1/6, 17%). Hyperphagia characterized nearly all individuals (5/6, 83%), contributing to progressive weight gain. Body mass index (BMI), which was initially within or below the normal range during early childhood, showed a clear upward trend with age. By mid-childhood, BMI exceeded the 95th percentile in all patients—except for one with dysphagia—and remained persistently elevated thereafter. At last follow-up, BMI values in the group ranged from 21.4 to 34.7 kg/m², with a median of 23.9 kg/m² (median z-score 2.7, range 0.9–3.6; *n* = 6). Where longitudinal data were available, BMI z-score trajectories demonstrated divergent patterns, with two patients showing an upward trend and two remaining relatively stable. No significant correlation between age and BMI z-score was observed (Spearman's *ρ*, *p* > 0.05). Height measurements were limited, precluding formal growth trajectory analysis; however, three patients (3/6, 50%) met the criteria for short stature (<3rd percentile).

#### Neurological problems

Nocturnal sleep disturbances and fatigue were the predominant neurological manifestations (6/6, 100%). Difficulties initiating sleep and frequent nighttime awakenings were the most common features of sleep disruption, occurring in relation to behavioral dysregulation, anxiety, mechanical ventilation, or absence of any other identifiable trigger. Ocular motility dysfunctions were observed in five patients (5/6, 83%), comprising divergent strabismus in four and convergence insufficiency in one. Strabismus was associated with refractive errors in two cases—one with isolated astigmatism and the other with combined astigmatism and hypermetropia; missing baseline data precluded determining if these were pre-existing. Concurrently, excessive daytime sleepiness (EDS) was also frequently reported (4/6, 67%). Regarding motor function, clumsiness was noted in four patients (4/6, 67%), and ataxia in two (2/6, 33%); however, assessment was limited by reduced ambulation across the population. Other neurological complications included sialorrhea (2/6, 33%)—secondary to severe dysphagia requiring percutaneous endoscopic gastrostomy in one case. Acute events were also documented, comprising status epilepticus in one patient during an intercurrent infection (1/6, 17%) and non-epileptic paroxysmal events with loss of consciousness of unclear etiology in two others (2/6, 33%). Autonomic dysregulation was found to involve multiple organ systems, representing a further dimension of neurological involvement. The most common complaints were decreased pain sensitivity (6/6, 100%), excessive sweating (5/6, 83%), and urinary incontinence (5/6, 83%). Thermoregulatory disturbances were another consistent finding (4/6, 67%), characterized by intermittent hyperthermia in three patients and alternating hyperthermia/hypothermia in the fourth. Cardiovascular and gastrointestinal autonomic signs were also prominent, including recurrent bradycardia (3/6, 50%), constipation (3/6, 50%), and fecal incontinence (3/6, 50%). Additional manifestations comprised abnormal pupillary reactivity (2/6, 33%), bilateral miosis (2/6, 33%), cold extremities (1/6, 17%) and paralytic ileus (1/6, 17%).

#### Behavioral and psychiatric problems

Emotional and behavioral disturbances were highly prevalent throughout the course of the disease. Mood alterations were observed in nearly all patients (5/6, 83%)—predominantly irritability, though one child exhibited a euphoric mood. Emotional dysregulation was equally pervasive, often precipitated by low frustration tolerance (5/6, 83%). Associated behavioral patterns included psychomotor agitation (5/6, 83%), hetero-aggressive behaviors (5/6, 83%), oppositional–defiant manifestations (5/6, 83%), and self-injury (2/6, 33%). Furthermore, progressive cognitive deterioration (5/6, 83%) emerged as a core feature, accompanied by language decline (4/6, 67%) and loss of previously acquired skills and autonomy (4/6, 67%). A tendency toward social withdrawal was also widely evident (5/6, 83%). Significantly, half of the population (3/6, 50%) struggled with adherence to treatment plans, including intolerance to life-saving devices, directly increasing mortality risks. Less frequent psychiatric features consisted of hyperactivity (1/6, 17%), anxiety symptoms (1/6, 17%), and a single case of obsessive–compulsive disorder (1/6, 17%).

#### Respiratory problems

In accordance with the diagnostic criteria for ROHHAD, central hypoventilation was a uniform finding across the cohort, characterized by recurrent prolonged apneas—most prominently during sleep—and associated episodes of persistent oxygen desaturation and cyanosis (6/6, 100%). Acute respiratory failure was the primary indication for initial hospitalization in four cases (4/6, 67%), including one instance of respiratory arrest necessitating emergent intubation. Obstructive sleep apnea, secondary to upper airway crowding and hypotonia, was identified in half of the patients (3/6, 50%). Respiratory infections (2/6, 33%) frequently exacerbated underlying hypoventilation, precipitating episodes of acute respiratory deterioration.

### Treatment approaches and management needs

To systematically address the diverse clinical challenges presented by these individuals, specific management strategies were adopted; these are detailed below according to clinical domain.

#### Endocrinological and metabolic problems

Therapeutic management included prompt initiation of levothyroxine for central hypothyroidism, resulting in good symptom control and tolerability. Among the three patients eligible for GH replacement, two developed clinically significant adverse effects at therapeutic doses. One experienced edema and rapid weight gain (+1.5 kg in one month) shortly after initiation (0.026 mg/kg/day) and again at a lower dose, leading to permanent discontinuation of therapy. The other developed persistent fluid retention, lethargy, hypotension, and hyponatremia during an intercurrent viral illness while receiving 0.07 mg/kg/day, requiring treatment suspension and later resumption at 0.02 mg/kg/day. The third did not start GH therapy due to family refusal. Central adrenal insufficiency was treated with hydrocortisone replacement (maximum dose 13.6 mg/m²/day), except in one patient with a partial form, who received only stress-dose glucocorticoid supplementation. Of the three individuals with AVP-D, two required long-term desmopressin therapy (maximum dosage 60 µg/day alternating with 45 µg/day). In the female patient with hypogonadotropic hypogonadism, sex-steroid replacement therapy led to the onset of menarche.

#### Neurological problems

Nocturnal sleep disturbances most often warranted active pharmacological management. Melatonin (2–5 mg/day) was used as first-line therapy in five cases, 5-hydroxytryptophan (maximum dose 100 mg/day) in one. Still, both approaches offered minimal or no benefit, urging the addition of further pharmacological agents. Medications of choice included hydroxyzine in two patients (0.5–1 mg/kg/day) and mirtazapine in three others (15–30 mg/day), both of which produced only transient improvement. Benzodiazepines (alprazolam, delorazepam, midazolam, lorazepam) were administered as second-line or as-needed treatments; however, they provided no sustained benefit and were frequently linked to lethargy and irritability, although concomitant polypharmacy complicated adverse-effect attribution. Evening doses of psychotropic medications were trialed for behavioral dysregulation, but their impact on sleep was inconsistent. EDS improved markedly following continuous nocturnal ventilation, with one exception. Motor symptoms, including ataxia, clumsiness, and fatigue, were addressed through targeted rehabilitation interventions. Three patients underwent physiotherapy programs focused on improving balance, gait, and functional mobility; one patient received neuro-psychomotor therapy to support overall functional development. Refractive errors were managed with corrective lenses. Regarding autonomic dysfunction, hyperthermia was treated with paracetamol as needed, although with variable response. Management of constipation involved dietary modifications and laxatives (e.g., macrogol), whereas excessive sweating was addressed with topical antiperspirants. Toilet training was attempted for urinary incontinence with limited success, partly due to behavioral disturbances and neurodevelopmental impairments. Transdermal scopolamine (0.3 mg/24 h release) achieved effective sialorrhea control in the single instance it was employed.

#### Behavioral and psychiatric problems

Behavioral disturbances were managed primarily through pharmacological interventions, mainly atypical antipsychotics, including olanzapine (maximum dosage 10 mg/day), risperidone (maximum dosage 2.5 mg/day), and aripiprazole (maximum dosage 10 mg/day) as first-line treatments. Subsequent switches between first-line agents were required in multiple cases due to poor tolerability or limited efficacy. Haloperidol (2.5 mg/day) and lurasidone (dosage unknown) were employed as additional second-line monotherapies. In four patients with severe self- or hetero-aggressive behaviors, polypharmacological regimens were necessary, combining typical antipsychotics with a more substantial sedative effect—promazine (20–150 mg/day), clotiapine (30–70 mg/day), or chlorpromazine (62.5 mg/day)— with benzodiazepines (delorazepam, lorazepam). One patient received a particularly complex regimen including risperidone, promazine, methylphenidate, valproate, mirtazapine, and cannabidiol oil, yet improvements were marginal. Overall, pharmacological therapy yielded limited efficacy, failing to prevent progressive behavioral deterioration or prevent attempts to remove life-saving devices in cases of poor treatment adherence. Adverse effects included extrapyramidal symptoms with risperidone (1/1), hallucinations with methylphenidate (1/1), and hyperthermia in one patient receiving clotiapine and chlorpromazine, with uncertain attribution to a specific agent. Additional effects included paradoxical behavioral worsening with aripiprazole (1/3), increased appetite with chlorpromazine (1/1), and excessive sedation with clotiapine (1/1). Mechanical restraint was sometimes required to support respiratory management. Severe behavioral dysregulation resulted in repeated emergency admissions; safe clinical management necessitated dexmedetomidine sedation in one patient. Early in the disease course, before behavioral escalation restricted intervention options, psychoeducational therapy was implemented with modest clinical benefit.

#### Respiratory problems

Continuous ventilatory monitoring and timely initiation of assisted ventilation were essential components of care. At referral to our PPC center, three patients were already receiving invasive mechanical ventilation via tracheostomy. Two, initially managed with non-invasive ventilation, rapidly progressed to tracheostomy. The remaining patient had not initiated any consistent ventilatory support owing to proportionality-of-care considerations; they were later unable to tolerate non-invasive ventilation, whereas tracheostomy was deemed clinically inappropriate because of severe behavioral dysregulation and high risk of accidental decannulation. Among the five tracheostomized patients*,* the median interval between the onset of the presenting symptom and tracheostomy placement was 0.6 years (range 0.3–10.4; *n* = 5), with the most extended interval reflecting an initial parental refusal of the procedure. Ventilator settings were individualized according to disease severity and periodically adjusted in response to gas-exchange parameters and sleep-related variables. Invasive ventilation was delivered predominantly with pressure-controlled or pressure-support modes with a mandatory backup respiratory rate, using inspiratory pressures ranging from 10 to 28 cmH₂O, positive end-expiratory pressure (PEEP) of 3–5 cmH₂O, and tidal volumes of 5–9 mL/kg. When feasible, non-invasive ventilation was provided using pressure-control or volume-targeted modes*.* Respiratory infections were managed with supportive therapy and targeted antibiotics when a bacterial etiology was identified; in these cases, hospitalization was frequently required due to the substantial risk of progressive respiratory compromise and to provide caregiver relief.

#### Other complex healthcare needs

**Figure 1:**
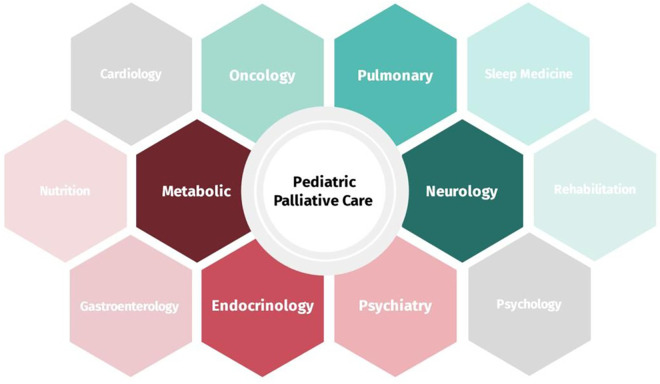
Interdisciplinary care model of ROHHAD syndrome.

**Figure 2:**
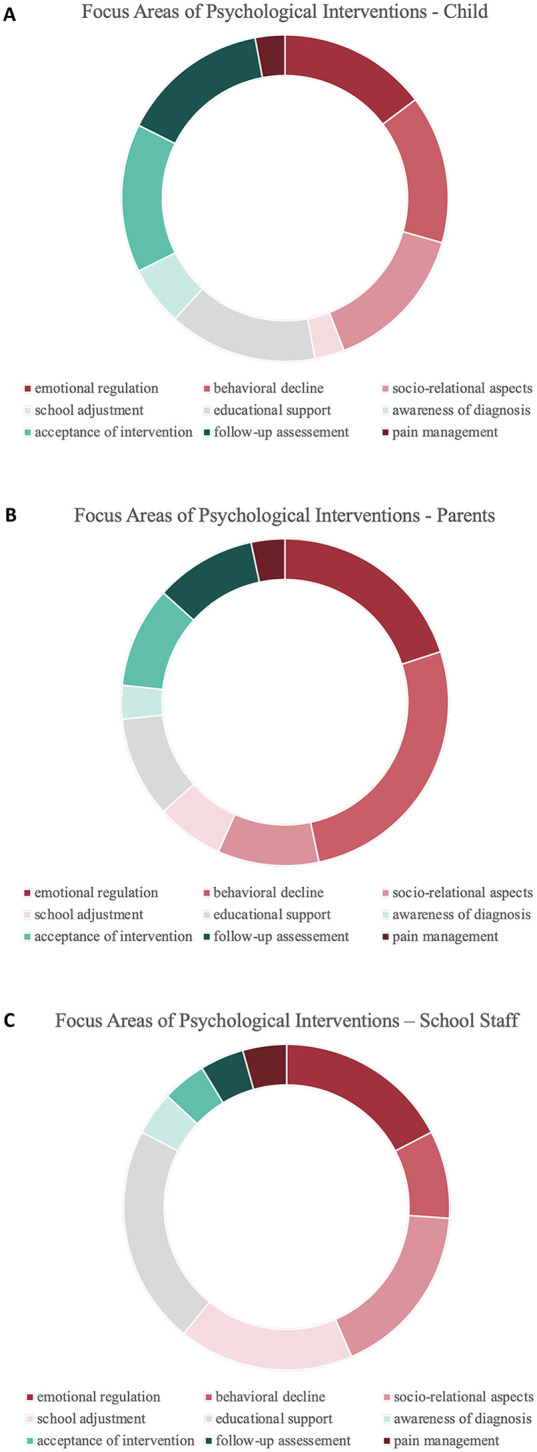
Primary domains of psychological intervention across children **(A)**, parents **(B)**, and school staff **(C)**.

PPC was integrated promptly following the onset of presenting symptoms, with a median time of 0.8 years (range 0.2–1.7; *n* = 6). It involved coordinated, interdisciplinary care that addressed a range of complex needs, including clinical management, psychosocial support, and collaboration with educational and community services. The specific components of the PPC involvement are detailed in Table 3. Figure 1 illustrates the multidisciplinary network implicated in the management of ROHHAD. This interdisciplinary alignment was supported by regular meetings to update individualized care plans in response to evolving clinical needs. Interventions were provided during hospitalizations across multiple settings and at home; however, one case was ineligible for home-based support due to out-of-region residency. PPC ward admissions ranged from 3 to 50 per case, highlighting significant heterogeneity in clinical trajectories, care intensity, and disease duration. Telephone and telemedicine consultations were pivotal in maintaining direct communication, facilitating ongoing symptom surveillance, and ensuring the timely adjustment and reinforcement of therapeutic continuity across professional teams. Nursing care reflected the inherent complexity of the condition, requiring a delicate balance between ventilatory technical skills and strategies for managing oppositional behaviors to ensure safe respiratory support in both hospital and home settings. Families received training to recognize early signs of respiratory compromise, manage basic equipment-related issues, and execute emergency protocols. Nursing staff facilitated this transition through targeted instruction and emotional support. Key challenges involved adapting ventilatory interfaces to specific anatomical needs, preventing complications such as skin breakdown, and mitigating the psychosocial impact of behavioral dysregulation. Psychological support was implemented for four subjects, addressing domains such as diagnostic awareness, emotional regulation, and socio-relational aspects; in the remaining two cases, these interventions were deferred due to cognitive limitations that precluded meaningful participation. Parental participation was sustained throughout, either individually or in pairs. For those enrolled in school, intersectoral coordination meetings facilitated collaborative planning and promoted educational and social inclusion, with interventions targeting school adjustment, peer interactions, and staff support. Further details regarding psychological interventions are provided in Figure 2. Advance care planning (ACP) was completed for all six patients and reviewed regularly, providing a structured framework to clarify goals of care and guide decision-making in complex clinical situations. Respiratory arrest was the primary cause of death (3/4, 75%), with events occurring abruptly and most often in the context of inadequate ventilation related to poor treatment adherence. Three patients died at home; two had advance care plans limiting maximal interventions. The fourth patient died in the hospital from complications of sepsis under a shared care plan that included a do-not-resuscitate order.

**Table 3:** Components of the PPC intervention.

Type	Median (range)
Admissions to pediatric hospice	11 (3–50)
Multidisciplinary clinical team meetings	12 (4–29)
Home care visits	8 (0–26)
School-based care coordination meetings	3 (0–14)
Telephone and telemedicine-supported contacts	42 (3–110)

## Discussion

ROHHAD syndrome encompasses a broad constellation of multisystemic disturbances that may emerge at different stages and progress unpredictably (4, 7). Such variability often delays recognition and diagnosis, impeding the timely initiation of targeted management. Even with early identification, ROHHAD remains difficult to treat, as many manifestations are refractory to standard therapies, necessitating a lifelong, interdisciplinary approach to care (5). In this retrospective analysis, we aim to refine clinical patterns, characterize therapeutic needs, and evaluate outcomes in order to provide a more comprehensive framework to address this rare and severe condition.

Rapid-onset obesity typically serves as the sentinel event, appearing predominantly between 3 and 4 years of age, though late-onset presentations (up to 8–9 years) have been documented (1, 2, 11). Our findings align with this temporal distribution, emphasizing that although early-childhood emergence is characteristic, clinicians must maintain high surveillance for later presentations and implement comprehensive screening strategies coupled with rigorous longitudinal assessments for evolving hypothalamic, respiratory, and autonomic abnormalities. Indeed, evidence suggests that these secondary manifestations may emerge months or even years following the initial weight gain, making early recognition sometimes particularly difficult (1, 12). The diagnostic delay observed in our series is consistent with prior reports, in which diagnosis was frequently deferred by more than one year due to the gradual evolution of clinical features (4, 5, 11). Such delays pose a significant prognostic risk, as respiratory and autonomic instability can progress subclinically, thereby increasing the likelihood of acute decompensation. These patterns underscore the importance of maintaining a heightened index of suspicion and establishing early, specialized referral pathways, even when presenting features are partial or subtle.

The extensive impact of hypothalamic dysfunction in ROHHAD highlights the hypothalamus's critical role in homeostasis. Beyond neuroendocrine abnormalities, disturbances in metabolic, fluid-electrolyte, and autonomic regulation are common (13, 14). Endocrine involvement is typically multiaxial, with various hormonal defects clustering within individual patients (1, 5). In our study, central hypothyroidism was the most common abnormality—and the one for which therapeutic control was most easily achieved. GH deficiency was present in most patients who underwent endocrine evaluation, at a frequency notably higher than the ∼25% reported in the largest systematic review to date (7). In two out of three cases, significant adverse events emerged during GH therapy—including edema, fluid retention, hypotension, and hyponatremia—requiring dose reduction or discontinuation. Together, these observations suggest a potential susceptibility to fluid-balance instability and emphasize the need for cautious, individualized GH use with close monitoring. Adrenal axis impairment included partial forms and cases precipitated by corticosteroid exposure, aligning with earlier descriptions (6). Electrolyte instability was also recurrent, with several patients exhibiting persistent or fluctuating sodium disturbances. Emerging evidence suggests that dysnatremia in ROHHAD may stem from extensive hypothalamic impairment affecting thirst perception and osmoregulatory signaling, rather than an isolated defect in vasopressin secretion (9, 15), better explaining the variability of sodium abnormalities in affected children. One notable metabolic aspect was the presence of hepatic steatosis in some cases despite normal liver enzyme levels, underscoring the limited sensitivity of transaminases as screening markers and supporting the incorporation of routine hepatometabolic assessment into standard clinical care. Collectively, these results elucidate the multisystemic and progressive nature of hypothalamic involvement, identifying it as the primary driver of clinical instability in ROHHAD—as opposed to a purely endocrine dysfunction—validating periodic reevaluations to ensure comprehensive assessment and prompt diagnostic testing.

Autonomic dysregulation is likewise a significant and highly variable trait of ROHHAD (4, 16). Bradycardia is one of the most salient manifestations, occurring unpredictably even during periods of apparent physiological stability (1, 11). In our cohort, decreased pain sensitivity and excessive sweating were the most prominent dysautonomic features, reflecting substantial involvement of sudomotor and visceral pathways (16). Sphincter dysfunction, thermal instability, and gastrointestinal dysmotility persisted over time, increasing caregiver burden and complicating daily care, with few effective therapeutic options available.

Central hypoventilation emerges as a major and potentially life-threatening manifestation of ROHHAD. Importantly, early respiratory evaluations may be normal, as hypoventilation can arise later in the disease course (11, 17). Longitudinal studies demonstrate that some patients initially present with obstructive sleep apnea or subtle sleep-disordered breathing, evolving afterward to nocturnal and eventually daytime hypoventilation (11, 18). Progressive blunting of chemosensitivity to hypercapnia and hypoxia has also been described, primarily in small physiologic studies and case series, and is thought to contribute to episodes of silent hypercapnia and increased cardiorespiratory instability (1, 19). In parallel, limited polysomnographic and observational data suggest that ROHHAD may be associated with disrupted sleep architecture and diminished arousal responses, further impairing the ability to respond effectively to worsening gas exchange and increasing the risk of respiratory decompensation (20–22). In our cohort, acute respiratory insufficiency was the primary cause for emergency presentation and intensive care admission. These events progressed rapidly, with most individuals requiring tracheostomy within the first year after the initial presenting symptom. Ventilator settings were consistent with a lung-protective strategy, using moderate inspiratory pressures and relatively low levels of PEEP to ensure adequate ventilation while limiting barotrauma and hemodynamic effects. Such findings highlight the aggressive trajectory that can accompany ROHHAD and the substantial management challenges it introduces, indicating that even patients who appear clinically stable require close ventilatory surveillance and proactive planning to ensure timely escalation of respiratory support. Management implies tailored ventilatory titration, informed by the continuous monitoring of respiratory mechanics, gas exchange, polysomnographic breathing patterns, and the broader clinical evolution.

Sleep disturbances were another significant issue among the individuals we evaluated, with limited response to both pharmacological and non-pharmacological measures and a substantial impact on daily functioning and quality of life (QoL). EDS—though not quantified through formal psychometric or objective assessment—seemed to manifest as a consequence of fragmented, non-restorative nocturnal sleep rather than indicating a primary hypersomnolence disorder. Optimization of nocturnal ventilatory support was generally associated with enhanced daytime alertness, substantiating the importance of rigorous nighttime respiratory management in these patients. Sporadic reports have described hypersomnolence-like features in ROHHAD, including documented hypocretin-1 deficiency in a few patients, suggesting that actual overlap with central hypersomnolence syndromes may be present but is likely rare (23, 24). Given the paucity of systematic sleep–wake assessments in this condition, further research is warranted to determine whether a discrete hypersomnolence phenotype exists or is predominantly driven by nocturnal respiratory alterations. The profound burden of these symptoms—often underestimated in the absence of rigorous assessment and monitoring—dictates the necessity of recurrent polysomnographic evaluations. Within the broader neurological profile of our cohort, motor impairment emerged as a recurrent finding, suggesting an under-recognized facet of the ROHHAD phenotypic spectrum. While not classified as a hallmark of ROHHAD (1, 5), these deficits likely originate in the synergistic burden of obesity, fatigue, and autonomic dysfunction, compounded by an intrinsic central neurological disruption. In view of the substantial threat to mobility and posture, early physiotherapy and structured rehabilitation programs may prolong functional independence and autonomy in daily activities.

ROHHAD has been associated with a broad spectrum of psychiatric and behavioral manifestations (4, 25, 26). In our population, these symptoms were more frequent and severe than previously reported and, together with central hypoventilation, constituted a major challenge in clinical management, while also being observed in conjunction with reduced QoL and poorer prognosis. Indeed, behavioral problems were particularly refractory to pharmacological treatment, often requiring complex polypharmacy, which in turn increased the risk of adverse effects and further complicated the therapeutic approach. In this context, emotional dysregulation and poor tolerance of life-sustaining devices frequently resulted in recurrent device removal or interruption of ventilatory support, contributing to repeated hospitalizations and co-occurring with earlier mortality. Importantly, cognitive involvement may represent a further extension of this neurobehavioral phenotype and a marker of disease evolution (27), with impairment potentially affecting treatment adherence and respiratory stability and thereby contributing to a self-perpetuating cycle of clinical decline. Although not traditionally considered core features of the disease, our evidence indicate that neuropsychiatric symptoms are recurrent and substantially disabling across patients, warranting their recognition as integral and primary components of the ROHHAD phenotype and surpassing the definition of merely comorbid conditions. Another aspect worth noting in our study group was the high prevalence of neurodevelopmental disorders, standing in contrast to earlier literature describing typically normal development at baseline (2, 4, 6). This pattern raises questions regarding whether such alterations may represent early or evolving manifestations of central nervous system impairment in ROHHAD rather than incidental or secondary features of the disease.

Tumor association is well documented in ROHHAD, with NETs—most commonly ganglioneuroma, ganglioneuroblastoma, or neuroblastoma—identified in approximately 40%–56% of reported cases (7, 28). When present, these tumors are typically diagnosed within two years of obesity onset and are often located in the thoracic or abdominal cavities (4, 5). Notably, some lesions remain clinically silent and are detected only through systematic imaging surveillance (5). Initial screening failed to detect NETs in our cohort; however, it remains uncertain to what extent these negative findings reflect a true absence of neoplastic lesions as opposed to limitations in imaging sensitivity and the timing of assessments. Beyond these considerations, recent data support the hypothesis of phenotypic heterogeneity within the ROHHAD spectrum, in which tumor-associated cases (ROHHAD-NET) represent one clinical subtype, potentially distinct in pathophysiology from tumor-negative presentations. Emerging evidence, including the identification of anti-ZSCAN1 as a highly specific biomarker, points to an immune-mediated mechanism in ROHHAD. In individuals with associated tumors, the condition has been proposed to reflect a paraneoplastic process triggered by autoimmune responses to neuroblastic tumor antigens, resulting in hypothalamic and autonomic dysfunction (10, 29). Conversely, tumor-negative cases may arise from alternative autoimmune or epigenetic drivers, or from an occult neoplasm that initiated a paraneoplastic cascade prior to clinical detection. Accordingly, routine screening at diagnosis is warranted, yet the absence of a detectable tumor does not exclude ROHHAD or reduce the need for ongoing surveillance.

As ROHHAD syndrome lacks a defined monogenic etiology (6, 30, 31), genetic testing is primarily indicated to exclude clinically overlapping disorders, particularly Congenital Central Hypoventilation Syndrome (CCHS) and syndromic obesity (5). Investigations within our cohort were highly heterogeneous, reflecting the lack of a standardized diagnostic algorithm. Their non-diagnostic yield is in line with current literature, which increasingly points away from a primary monogenic driver in favor of more complex pathogenic mechanisms (29).

Historically, the management of ROHHAD has been exclusively supportive, focusing on life-sustaining interventions for central hypoventilation, hormone replacement for endocrine deficiencies, and tumor surveillance. However, this symptom-based approach does not address the progressive hypothalamic dysfunction characterizing the syndrome. The hypothesis of ROHHAD as a potential immune-mediated disorder has prompted the exploration of possible mechanistic, disease-modifying strategies. In this context, recent reports have described the use of targeted immunomodulatory therapies, including high-dose cyclophosphamide, rituximab, and intravenous immunoglobulin (IVIG), aimed at attenuating neuroinflammation during an early, potentially reversible phase of disease in an attempt to preserve hypothalamic function (25, 32, 33). In our series, management remained predominantly supportive, and advanced immunomodulatory approaches were not systematically implemented, partly due to the unavailability of anti-ZSCAN1 antibody testing, which precluded immune–phenotype correlation. Although three patients received empirical IVIG prior to formal diagnosis, systematic evaluation of immunomodulatory efficacy was not undertaken, and longitudinal outcomes will be reported separately.

PPC was essential in addressing the complex, multifactorial, and evolving care needs of children with ROHHAD, providing coordinated, patient-centered interventions across multiple domains of care. ACP ensured alignment with patient and family preferences throughout the course of the disease while adapting to evolving needs and thereby preserving care continuity. Indeed, early incorporation of ACP is particularly relevant in the management of this syndrome, as uncertainty around disease progression may challenge both medical and family expectations. Psychological support played a crucial integrative role. In ROHHAD, the burden on families extends beyond caregiving demands, encompassing profound psychological distress, social and economic strain, and complex bioethical challenges related to life-sustaining treatments and proportionality of care. School withdrawal and social isolation due to cognitive and behavioral decline can significantly aggravate family strain, as the loss of this psychosocial anchor shifts the entire burden of care and socialization to the domestic environment. Consequently, proactive integration with social services and the establishment of personalized educational pathways are essential. Schools must serve as vital conduits for social connectivity, ensuring that inclusive engagement remains a priority even for children with complex conditions like ROHHAD (34). Indeed, these multidimensional dynamics often destabilize family functioning and well-being, which in turn may further compromise treatment adherence. The resulting trajectory emphasizes the fundamental importance of comprehensive, continuous psychosocial care, particularly during phases of clinical and behavioral deterioration. Lastly, nursing management proved exceptionally difficult across both home and hospice settings. In such complex healthcare conditions, nursing efficacy is predicated not only on technical proficiency but on an expanded role involving caregiver training, advocacy, and holistic family-centered interventions (35). Ultimately, these demands require an integrated nursing paradigm for pediatric patients with ROHHAD that balances clinical precision with continuous care coordination and adaptation to their evolving needs.

In conclusion, this study outlines the significant clinical variability of ROHHAD and marks the centrality of interdisciplinary collaboration and dynamic, context-responsive interventions across clinical, educational, and community settings. Transitioning support beyond the hospital environment is vital to ensure continuity of care for both the patient and the family. Important gaps in understanding remain, for which shared efforts with systematic data collection will be essential to refine current approaches and improve long-term outcomes for affected children.

### Limitations

Main limitations include the small cohort size (*n* = 6), consistent with the rarity of ROHHAD, and the retrospective nature of data collection, which depended on variable clinical documentation in the absence of a standardized and comprehensive assessment protocol, restricting generalizability and precluding formal statistical analysis. Moving forward, the implementation of longitudinal total-body imaging and integration with autoimmune assays—specifically for anti-ZSCAN1 autoantibodies—may help expediting clinical detection, improving stratification for targeted therapies, and better defining the primary drivers of the disease. Furthermore, contributing to collaborative data-sharing networks is vital for pooling clinical insights and unifying management across centers in order to overcome the limitations of disease rarity.

## Data Availability

The original contributions presented in the study are included in the article/Supplementary Material, further inquiries can be directed to the corresponding author.
