# Supplementary material for: ROHHAD syndrome: an interdisciplinary perspective
Source: Front Pediatr. 2026 Jun 3;14:1777286. doi: 10.3389/fped.2026.1777286 (PMC13273399; doi:10.3389/fped.2026.1777286)
Supplement: Supplementary file 1 [file Table1.pdf]

Supplementary Table S1 – Summary of radiological and genetic investigations performed for each individual patient

	P1	*P2*	*P3*	*P4*	*P5*	*P6*
**Neuroimaging and Tumor Screening**						
Brain MRI	+	+	+	+	+	+
Abdominal ultrasound	+	+	+	+	+	+
Thoracic CT					+	
Thoracoabdominal CT	+					
Whole-body CT				+		
Whole-Body FDG PET/CT		+	+			
**Genetic Characterization**						
Karyotyping	+		N/A			+
Array-CGH			N/A		+	+
PWS/AS methylation analysis	+		N/A		+	+
Fragile X (FMR1) analysis			N/A			+
Targeted *PHOX2B* analysis (Sanger)	+	+	N/A			+
Mitochondrial DNA (mtDNA) sequencing		+	N/A			
Whole Exome Sequencing (WES)*		+	N/A	+	+	+

Legenda: AS: Angelman syndrome; CGH: comparative genomic hybridization; FDG: [^{18}F] fluorodeoxyglucose; N/A: not available; PWS: Prader-Willi syndrome

*WES analysis included targeted virtual panel filtering for PHOX2B and P4HTM to exclude CCHS and P4HTM deficiency, respectively.
