# Supplementary material for: ROHHAD syndrome: an interdisciplinary perspective
Source: Front Pediatr. 2026 Jun 3;14:1777286. doi: 10.3389/fped.2026.1777286 (PMC13273399; doi:10.3389/fped.2026.1777286)
Supplement: Supplementary file 2 [file Table2.pdf]

Supplementary Table 2 – Individual distribution of clinical features across the study cohort

	Frequency, %	*P1*	*P2*	*P3*	*P4*	*P5*	*P6*
**Endocrinological problems**							
Obesity	6/6, 100%	+	+	+	+	+	+
Hypogonadotropic hypogonadism	2/2, 100%	N/A	N/A	+	N/A	N/A	+
Central hypothyroidism	5/6, 83%	+	+	+	+	+	
Growth hormone deficiency	3/4, 75%	+	+	+	N/A	N/A	
Adrenocortical insufficiency	4/6, 67%		+	+	+	+	
Hyperprolactinemia	3/5, 60%	+	+		N/A		+
Sodium disturbances	3/5, 60%		+	N/A	+	+	
Arginine vasopressin deficiency	3/6, 50%			+	+	+	
Short stature	3/6, 50%	+	+				+
**Metabolic problems**							
Hepatic steatosis	4/6, 67%	+	+	+	+		
Insulin resistance	2/4, 50%	+		+	N/A	N/A	
Impaired glucose tolerance	1/6, 17%	+					
Dyslipidemia	1/6, 17%		+				
Hepatomegaly	1/6, 17%					+	
Cholelithiasis	1/6, 17%		+				
Cholestasis	1/6, 17%				+		
**Neurological problems**							
Nocturnal sleep disturbances	6/6, 100%	+	+	+	+	+	+

Fatigue	6/6, 100%	+	+	+	+	+	+
Ocular motility disorders	5/6, 83%	+	+		+	+	+
Excessive daytime sleepiness	4/6, 67%		+	+	+		+
Motor clumsiness	4/6, 67%		+		+	+	+
Ataxia	2/6, 33%		+				+
Sialorrhea	2/6, 33%				+		+
Dysphagia	1/6, 17%						+
Seizures	1/6, 17%						+
Autonomic dysfunction							
Decreased pain sensitivity	6/6, 100%	+	+	+	+	+	+
Excessive sweating	5/6, 83%	+	+	+	+	+	
Urinal incontinence	5/6, 83%	+	+	+		+	+
Hyperthermia	4/6, 67%		+	+	+		+
Bradycardia	3/6, 50%		+	+	+		
Constipation	3/6, 50%			+	+		+
Fecal incontinence	3/6, 50%	+	+			+	
Abnormal pupillary reactivity	2/6, 33%		+		+		
Bilateral miosis	2/6, 33%					+	+
Hypothermia	1/6, 17%		+				
Cold extremities	1/6, 17%			+			
Paralytic ileus	1/6, 17%				+		
**Behavioral and Psychiatric problems**							
Mood disorders	5/6, 83%	+	+		+	+	+

Recurrent psychomotor agitation	5/6, 83%	+	+		+	+	+
Frustration intolerance	5/6, 83%	+		+	+	+	+
Hetero-aggressive behavior	5/6, 83%	+		+	+	+	+
Oppositional–defiant behaviors	5/6, 83%	+	+		+	+	+
Neurocognitive dysfunction	5/6, 83%	+	+		+	+	+
Social isolation	5/6, 83%	+	+		+	+	+
Language decline	4/6, 67%	+	+		+	+	
Loss of skills and autonomy	4/6, 67%	+	+		+		+
Intolerance to life-saving devices	3/6, 50%	+			+	+	
Self-injurious behavior	2/6, 33%	+					+
Anxiety	1/6, 17%		+				
**Respiratory problems**							
Central hypoventilation	6/6, 100%	+	+	+	+	+	+
Recurrent oxygen desaturation with cyanotic episodes	6/6, 100%	+	+	+	+	+	+
OSAS	3/6, 50%		+	+			+
Recurrent respiratory infections	2/6, 33%	+			+		
**Other**							
Hyperphagia	5/6, 83%	+	+	+	+	+	
Non-epileptic paroxysmal events	2/6, 33%				+	+	
NET*	0/6, 0%						

Legenda: OSAS: obstructive sleep apnea syndrome; N/A: not available; NET: neuroendocrine tumor.

Note: N/A denotes the absence of serial or comprehensive biochemical assessment.

*As assessed by the available imaging modalities.
